# Supplementary material for: A data management infrastructure for the integration of imaging and omics data in life sciences
Source: BMC Bioinformatics. 2022 Feb 7;23:61. doi: 10.1186/s12859-022-04584-3 (PMC8822871; doi:10.1186/s12859-022-04584-3)
Supplement: Supplementary file 1 — Additional file 1: Fig. S1. Diagram of the unified metadata model, depicting the coupling between the underlying models and the cardinality relationship between metadata entities. (A) The hierarchical metadata model used by qPortal to describe the experimental design of a research project, containing general information of sample biology. (B) The hierarchical metadata model used by OMERO, focused on describing imaging data. Fig. S2. The file size distribution of the synthetic project and benchmark test. (A) The distribution of file sizes for the synthetic project per modality. The X-ray CT data was sampled from the LiTS [19] training dataset (130 tomograms), the H&E stained histology images were sampled from the training dataset of MoNuSeg [20] (30 images), and the genomics dataset was obtained from the 1000 genomes project [21] (260 paired-end illumina whole genome sequencing datasets). (B) Results of the benchmarking test for data registration, showing sequential registration times for imaging data (x-ray CT and histology images) and genomics sequencing data (WGS) for an increasing number of patients, where data for a single patient consists of 1 x-ray CT, 20 histology images, and 4 fastq files (paired-end data for cancer and healthy tissue, respectively). The curves follow the mean values of 4 registration runs (black dots). [file 12859_2022_4584_MOESM1_ESM.pdf]

Supplementary material:
A data management infrastructure for the integration of imaging and omics data in life sciences

Luis Kuhn Cuellar[1], Andreas Friedrich[1], Gisela Gabernet[1], Luis de la Garza[1], Sven Fillinger[1], Adrian Seyboldt[1], Tobias Koch[1], Sven zur Oven-Krockhaus[2], Friederike Wanke[2], Sandra Richter[2], Wolfgang M. Thaiss[3], Marius Horger[4], Nisar Malek[4], Klaus Harter[2], Michael Bitzer[4] and Sven Nahnsen[1,*].

[1]Quantitative Biology Center (QBiC), University of Tübingen, Tübingen, Germany

[2]Center for Plant Molecular Biology (ZMBP), University of Tübingen, Tübingen, Germany

[3]Department of Radiology, Diagnostic and Interventional Radiology, University of Tübingen, Tübingen, Germany

[4]Department Internal Medicine I, University of Tübingen, Tübingen, Germany

*Correspondence: Sven Nahnsen, sven.nahnsen@uni-tuebingen.de

Table of content

Unified and integrated metadata model 2

Performance analysis of data ingestion 3

Login details to the infrastructure 4

Unified and integrated metadata model

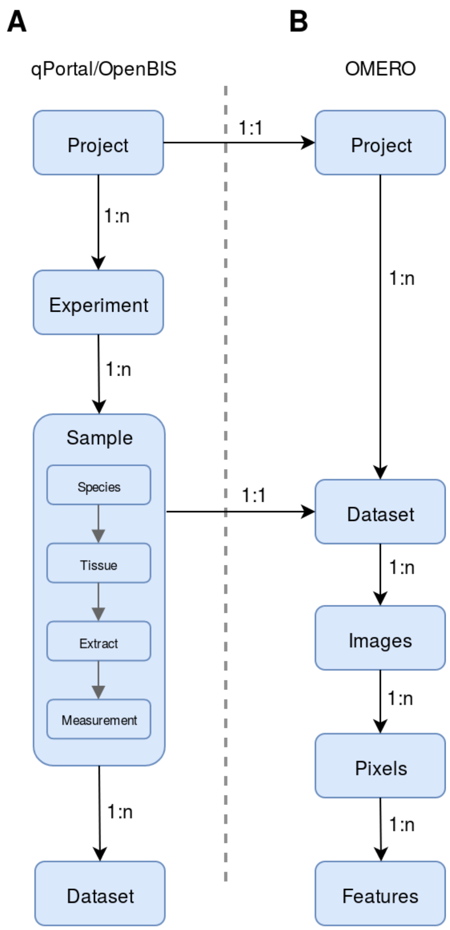

Fig. S1. Diagram of the unified metadata model, depicting the coupling between the underlying models and the cardinality relationship between metadata entities. **(A)** The hierarchical metadata model used by qPortal to describe the experimental design of a research project, containing general information of sample biology. **(B)** The hierarchical metadata model used by OMERO, focused on describing imaging data.

Performance analysis of data ingestion

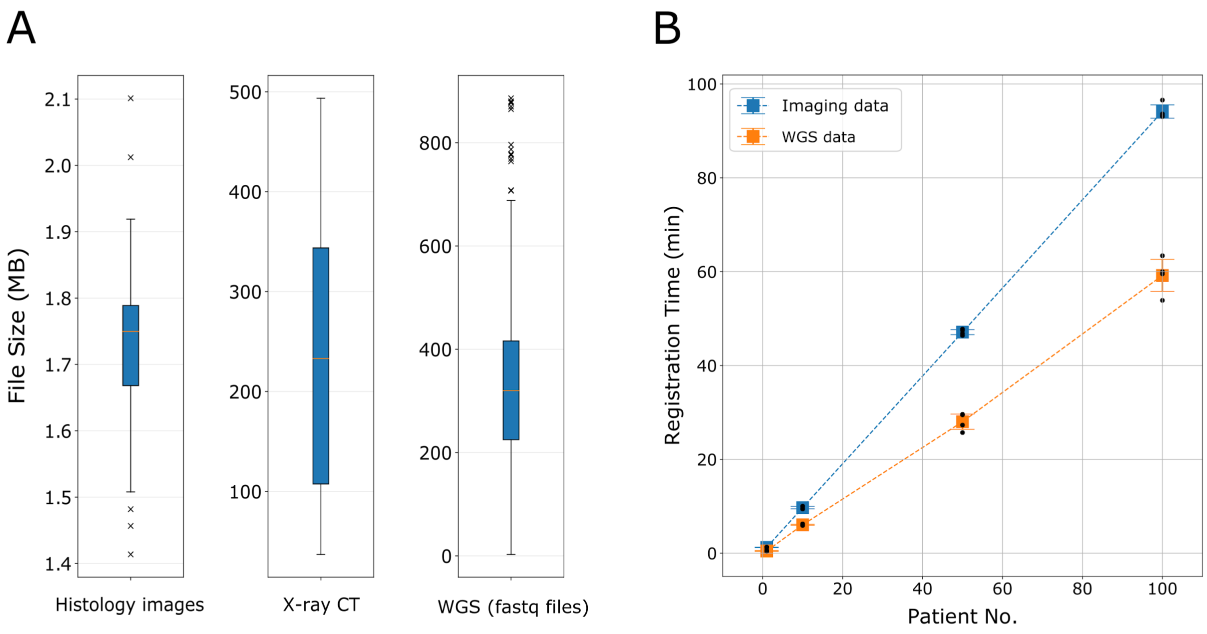

Fig. S2. The file size distribution of the synthetic project and benchmark test. **(A)** The distribution of file sizes for the synthetic project per modality. The X-ray CT data was sampled from the LiTS (Bilic et al. 2019) training dataset (130 tomograms), the H&E stained histology images were sampled from the training dataset of MoNuSeg (Kumar et al. 2017) (30 images), and the genomics dataset was obtained from the 1000 genomes project (Fairley et al. 2020) (260 paired-end illumina whole genome sequencing datasets). **(B)** Results of the benchmarking test for data registration, showing sequential registration times for imaging data (x-ray CT and histology images) and genomics sequencing data (WGS) for an increasing number of patients, where data for a single patient consists of 1 x-ray CT, 20 histology images, and 4 fastq files (paired-end data for cancer and healthy tissue, respectively). The curves follow the mean values of 4 registration runs (black dots).

Login details to the infrastructure

This account will only provide restricted access to the “synthetic clinical project”. To access the project (project code “QIMGT”), navigate to the “Project Management” tab, and select “Browser” or “Image Viewer”, to access the experimental design with omics data, and imaging data, respectively. The “Creator” application in the “Project Management” tab refers to the Project Wizard application and can be used to create new projects.

The account details to test the platform are the following:

```
Portal: http://qbic.life
User: qbcst01
Password: tgjz0ynmkdts
```
